# Digital storytelling across the life course: Protocol for a theory-based analysis

**DOI:** 10.1371/journal.pone.0332145

**Published:** 2026-06-25

**Authors:** Steven Hall, Michael Lang, Lauren Czarniewski, Cavan Fani, Ziyu Li, Kathleen Shearer

**Affiliations:** 1 Faculty of Medicine and Dentistry, Division of Geriatric Medicine, University of Alberta, Edmonton, Alberta, Canada; 2 Faculty of Nursing, University of Calgary, Calgary, Alberta, Canada; 3 Faculty of Nursing, University of Alberta, Edmonton, Alberta, Canada; University of Saskatchewan, CANADA

## Abstract

**Background:**

Digital storytelling (DST) combines first-person narratives with images, sound, and video to convey health experiences, empower under-represented voices, and foster empathy. Insight into how digital stories portray life-course influences remains scarce. Guided by the Life Course Health Development (LCHD) framework, this study protocol describes a planned theory-based analysis of 105 publicly available digital story videos from Alberta Health Services (AHS). We aim to (1) map representations of cumulative exposures, critical periods, and transitions across stories spanning childhood to late adulthood; and (2) generate guidance for using DST to strengthen partnerships among patients, caregivers, clinicians, and policymakers.

**Methods:**

We present our detailed methods for our planned study within this protocol using the Standards for Reporting for Qualitative Research checklist. Our team will review each video in AHS’ DST repository with a structured data collection form, capturing storyteller demographics, life-course stage, clinical context, LCHD constructs, and reflexive notes. Directed content analysis of our data collection forms will be performed in NVivo 14 with a hybrid coding approach: deductive codes to mirror core LCHD concepts and inductive codes to capture context-specific themes.

**Discussion:**

Insights from this study will guide clinicians’ life course dialogue, inform health systems’ investment in DST platforms for learning, and assist policymakers in embedding lived experience within equity agendas. The study will yield theoretically grounded recommendations for curating, analyzing, and mobilizing DST libraries across diverse health settings.

**Protocol Registration:** https://doi.org/10.17605/OSF.IO/C5R9T

## Background

Digital Storytelling (DST) is a genre of participatory media production where participants create and share short two to five minute films about important moments in their lives using images, videos, voiceovers, and music [1–4]. In heath contexts, DST captures personal narratives of health, illness, or care and can be co-created by patients and healthcare professionals or produced independently by individuals looking to share their own experiences [2,4,5]. These digital stories are used to educate, empower, and connect patients, healthcare professionals, and communities, serving as tools for learning, advocacy, and knowledge sharing [5–7]. Digital storytelling presents a compelling opportunity to share patient experiences, promote shared decision making, and increase awareness of experiences among patients, caregivers, healthcare professionals, and policymakers [3]. The method empowers underrepresented groups [7], supports culturally relevant health communication [6], and can also be a therapeutic outlet for storytellers [5]. Furthermore, DST has been used to honor local and cultural knowledge, and foster change [4–7].

The underlying psychosocial mechanisms of DST centers on its ability to foster understanding of oneself and others, stimulate emotional expression and engagement, and cultivate prosocial behaviors among individuals and groups [8]. Digital storytelling also allows individuals to process and share personal experiences, especially during or after crises, helping them make sense of events and emotions while keeping others informed and engaged [9,10]. For example, both Wilson et al. [10] and Laing et al. [11] used DST with pediatric oncology patients to help them explore their cancer experience and express their feelings and perspectives. As well, sharing digital stories can promote mutual understanding and empathy, such as in mental health contexts, by allowing people to communicate lived experiences and foster a sense of solidarity [12,13]. Intergenerational DST supports positive psychosocial development, especially for marginalized groups, by transmitting cultural knowledge, building resilience, and strengthening group identity [14].

The Life Course Health Development (LCHD) theoretical framework iterates that health is a dynamic trajectory that unfolds from pre-conception through to the end of life in response to continuously interacting biological, psychological, and socio-structural forces [15,16]. Central to LCHD is the premise that health trajectories are shaped and modifiable through patterned exposures embedded in time, place, and social relationships. By foregrounding temporal context, the framework helps researchers move beyond cross-sectional snapshots toward a deeper understanding of how early advantages or adversities can accumulate and how pivotal role changes can redirect developmental pathways [15].

This protocol describes a planned theory-based analysis of a library of digital stories archived from the Alberta Health Services (AHS) Patient and Family Storytelling initiative. The initiative was originally established as a patient engagement and organizational learning resource, designed to bring lived experience narratives into healthcare improvement efforts [17]. The initiative gave patients, families, and providers the opportunity to share first person accounts that could support reflection, empathy, education, and quality improvement across the health system [17]. The purpose of the library of digital stories extends beyond archiving stories to it functioning as a practice facing knowledge mobilization platform. Our study builds on this applied purpose by exploring how the repository can also serve as a corpus for theory-based analysis. Using the LCHD theoretical framework as a lens, we will explore how cumulative exposures, critical periods, and transitions can be presented in patient- and family-created digital stories. By applying a theory-informed analytic framework, we hope to illuminate patterns in how patients and families construct agency, assign meaning to clinical encounters, and advocate for system change as well as demonstrate how healthcare providers can use LCHD framework as a reflective tool to enhance their own learning when engaging with these digital stories. The LCHD model will allow us to analyze each digital story as a situated account of cumulative influences, critical periods, and life transitions.

## Methods

This study protocol was registered in OSF Registries on May 30, 2025 [18]. KS and SH conceptualized the study. SH prepared the protocol, and the study is led by KS. Our methods are reported herein using the Standards for Reporting Qualitative Research (SRQR) checklist [19], with some data items omitted or amended due to the nature of the study. We selected this checklist as a reporting guideline considering that our approach to analysis of the digital stories is qualitative in nature.

### Research questions

The two research questions guiding this work are: (1) “How do digital stories portray cumulative exposures, critical periods, and life-course transitions?” and (2) “What insights can this analysis offer for using digital storytelling to strengthen partnerships among patients, caregivers, clinicians, and policymakers?”

### Theoretical framework

We will use the Life Course Health Development (LCHD) model as a theoretical framework to guide our analysis and place the digital stories within stages of the life course: preconception, infancy, childhood, adolescence, early/ middle adulthood, and late/ older adulthood [15,16]. The LCHD model was created as a response to the limitations of traditional biomedical and biopsychosocial models, aiming to provide a more comprehensive understanding of how health develops over a lifetime [20]. It was developed by integrating research from biological, behavioral, and social sciences, and emphasizes the dynamic, interactive, and developmental nature of health across the lifespan [20]. The model is built on principles such as health development, unfolding, complexity, timing, plasticity, thriving, and harmony, which together describe health as a dynamic process shaped by interactions between biology and environment throughout life [21,15]. The model supports the design of interventions and policies that address social determinants of health and promote wellbeing across the lifespan [16,20–22].

The LCHD model recognizes that health trajectories are influenced by cumulative exposures, critical periods, and transitions at different life stages [15,23,24]. “Cumulative exposures” refers to the additive and synergistic effects of repeated or prolonged biological, psychosocial, and environmental influences across the lifespan. Examples include chronic neighborhood disadvantage, sustained caregiving stress, or long term adherence to health promoting behaviors [15]. Over time, these exposures become stacked, embedding risk or resilience into physiologic systems and shaping later function and disease vulnerability [15]. “Critical periods” in the LCHD are windows in which exposures exert disproportionately powerful effects on subsequent development. Examples include prenatal nutrition influencing metabolic setpoints or early childhood attachment shaping stress response [15]. Lastly, “transitions” denotes discrete role shifts or context changes, such as starting school, entering the workforce, receiving a chronic disease diagnosis, or moving into long term care [15]. These shifts recalibrate daily routines, social networks, and resource access. The three temporal concepts intersect with a broader set of LCHD constructs, which include: health development trajectories (the observable arc of functional capacity over time); timing and sequencing (when and in what order exposures and events occur); linked lives (the interdependence of individual trajectories within families and communities); plasticity (the capacity for adaptive change at biological and behavioral levels); agency and intentionality (how individuals and collectives act on opportunities to modify health pathways); and socio-ecological and inequity contexts (the structural conditions that stratify exposure patterns and resources).

### Research team and reflexivity

Our research team comprised of master’s-prepared registered nurse researchers (SH, KS) with backgrounds in pediatrics, general medicine, geriatrics and health services research as well as one team member (ML) who is PhD-prepared and the professional DST facilitator who supported the creation of all 105 stories being reviewed for this study. ML’s involvement is primarily to provide additional context to the primary findings of the story analysis. Having been involved in the creation of all the digital stories, he will be able to speak specifically to the initial project goals for each set of stories as well as the creation process for each story, which may inform the life course theory analysis. He will not be involved in the primary coding of the stories but will be involved in the analysis and writing stage. Team members also have experience accessing health systems as patients and family caregivers, allowing us to assess digital stories with an empathetic lens. Therefore, we are well-positioned to conduct this work because of our professional and personal understandings of health systems and healthcare provision. Moreover, our research backgrounds in qualitative and mixed methods work will enhance the quality and trustworthiness of the planned study.

### Data corpus

The collection of digital stories that will be analyzed comes from an initiative by Alberta Health Services (AHS) in Alberta, Canada. AHS hosts an online library of short, first person digital stories created in partnership with patients, family members, and healthcare professionals [17]. The initiative’s slogan is: “Real People. Real Experiences. Real Impact.” The publicly accessible repository currently features an array of digital stories narrated by individuals in the Early/Middle Adulthood and Late/Older Adulthood life stage with the stories encompassing every life stage, from fertility journeys (preconception) to children and adolescents navigating acute illness to older adults managing complex, chronic conditions. Contributors’ narratives span a wide spectrum of health experiences, including preventive care, emergency department encounters, long term rehabilitation, palliative care journeys, and family caregiving. Each story explores different social contexts, identities, and relational dynamics that shape health trajectories. As of May 2025, there are N = 105 digital stories published in the repository.

Currently, the repository is being maintained because it is a feasible way for managers, quality improvement professionals, and educators within AHS to embed patient stories and experiences directly into their quality improvement and educational activities. There are over 700 patient advisors with AHS. However, it is not always appropriate or feasible to have patient advisors attend specific meetings or educational opportunities. One of AHS’s main stated goals is to embed the patient voice into education and routine clinical practice context where appropriate [25]. The digital stories provide an opportunity to achieve this goal without being overly onerous on the patient advisor community. Combined, the digital stories have been viewed over 200,000 times, with the majority of those views happening within Alberta. Recently, it has become clear that the Patient Stories playlist has become a resource that has gone well beyond Alberta Health Services. Although the exact numbers of usage in specific contexts are not entirely clear, there has been significant viewership from across Canada and North America, represented in the YouTube viewership statistics. Also, there have been anecdotal reports of the stories being used in all academic nursing and medicine programs across Canada for teaching.

Originally, most of these stories were designed for health care providers to view and learn from, not for patients and families to learn from. However, it has become clear that patients and families also enjoy viewing these stories, and many patient advocacy groups or community support organizations also share the stories in different contexts. With the reorganization of Alberta Health Services recently, there has been a push to trim old programs and services and educational resources. However, there has been a very specific push to keep the patient stories repository as is because of its value to the organization and all the community partners that share these stories on a regular basis.

### Setting and context

Although the data corpus of digital stories is limited to Alberta, Canada, the provincial context offers several features that make the collection a broadly informative case for life course analysis. Alberta is home to approximately 4.9 million residents who are distributed across two major metropolitan centres (Calgary and Edmonton), dozens of mid-sized towns, and vast rural and northern regions [26]. The province has one of the youngest median ages in Canada, yet it also contains rapidly growing cohorts of older adults and a sizeable Indigenous population that includes First Nations, Métis, and Inuit communities [27,28]. Almost one-quarter of Alberta residents were born outside Canada, creating a mosaic of cultural and linguistic backgrounds [26]. This population heterogeneity with varied geographic and socioeconomic contexts indicates that health experiences captured in the digital stories encompass a wide range of exposures, critical periods, and transitions that could be transferable to many other settings.

### Ethical considerations

This study draws exclusively on digital stories housed in a publicly accessible AHS repository, where all storytellers provided informed consent for their narratives to be posted online. Because storytellers are intentionally identifiable by their first names within the videos, anonymity was not sought; however, their consent to be publicly identifiable was integral to their participation in the repository. As such, we have access to information that could identify individual storytellers during and after data collection, but only in the manner already made publicly available through the repository. Since this project involves secondary analysis of publicly accessible material and no interaction with storytellers or collection of new personal data, formal institutional ethics review is not required under current Canadian Tri-Council guidelines. As well, the only “new” data generated stem from our theory-based analysis using the Life Course Health Development model and do not compromise storyteller confidentiality or alter the original narratives. We received exemption from the University of Alberta’s Research Ethics Office from research ethics board review on August 19, 2025. Responsible data use will be maintained by citing the repository and presenting findings in aggregate form.

### Data collection

We will watch each digital story in the repository, taking field notes using a piloted data collection form. We collaborated in-person to complete the data collection form for n = 5 digital stories to ensure that expectations were clear, and the process was standardized. The remaining n = 100 digital stories will be divided between authors to watch and record field notes independently. When recording our field notes, we will use our pre-formulated analytic reflection prompts that accompany each LCHD construct (Table 1 in S4 File) as a guide for elements to record in the data collection form. Table 2 in S4 File presents our inter-rater reliability from when we were piloting the data collection form. We understand these initial results present a relatively low agreement for several LCHD constructs; however, we have reported the results here for transparency. For LCHD constructs which had a lower agreement rating, we discussed on how or why we made our decisions. KS and SH reviewed LCHD literature together in this discussion. What resulted was the decision to create analytic reflection prompts (presented in Table 1 in S4 File) to guide data collectors more clearly in their selection of LCHD constructs.

### Data collection instrument

Our unfilled data collection form is included in Supplementary File 1. The form is a fillable document in .PDF format. The form contains a space to record the digital story title, medical condition of storyteller, URL weblink of the digital story, reviewer name for follow-up if required, date of review, life course stage of both the storyteller and the persons receiving care (ranging from preconception to late/older adulthood), a checklist for LCHD constructs present in the digital story, and an open textbox to record narrative field notes.

### Data processing

A Microsoft Excel spreadsheet will be created to contain a list of every digital story in the repository. The spreadsheet will contain columns for: digital story title; URL (weblink); medical condition; and life course stage. Data for these columns will be extracted from the data collection forms. Each row will be numbered so that digital stories are assigned a numerical identifier. The data collection form files will be named “DSALC Data Collection_Digital Story X” with ‘DSALC’ being the abbreviated study title (“Digital Storytelling Across the Life Course”) and ‘X’ being a placeholder for the numerical identifier in the Microsoft Excel spreadsheet. These files will then be imported into NVivo 14 [29] for subsequent data analysis.

### Data analysis

We will employ a hybrid approach to coding and conduct a directed content analysis [30]. First, we will use a deductive codebook (Table 1 in S4 File) anchored to core LCHD constructs: health trajectory, sensitive/critical periods, cumulative risk and protective factors, plasticity and adaptive capacity, linked lives and intergenerational effects, and timing and environmental context [15,16]. These constructs are further described in Table 1 in S4 File under the “Digital Story Elements” column. The completed data collection forms will identify which of the deductive (‘top-down’) code groups are applicable in each digital story. We will subsequently analyze the recorded field notes from the data collection forms in NVivo 14 [29] and create inductive (‘bottom-up’) codes to capture emergent patterns, specific to Alberta and the Canadian healthcare system (e.g., encounters with universal healthcare, Indigenous Truth and Reconciliation calls, or the expansion of virtual care during the COVID-19 pandemic). The codes created inductively will be sorted under the overarching deductive codebook categories. The data analysis process will be led by KS, with assistance from three undergraduate nursing student research trainees. The undergraduate trainees will review literature on content analysis, be trained on NVivo software, and code the collected data. Team meetings will allow for check-ins with the trainees and debriefing on the coding process to mitigate potential bias. All analysis will then be reviewed and finalized by KS.

Because storytellers are identifiable in the public repository, we will take care to minimize interpretive and reputational harm in our secondary analysis. Our analysis will emphasize cross-story patterns rather than judgments about individual storytellers, and we will avoid speculative interpretations not clearly supported by the narrative content. Findings will be reported in aggregate, with identifying details used sparingly and only when necessary for analytic clarity. Ongoing team discussion during analysis will further support respectful, evidence-grounded interpretation of the stories.

### Techniques to enhance trustworthiness

We will use strategies to address the four criteria outlined by Lincoln and Guba [31] to enhance trustworthiness: credibility, dependability, confirmability, and transferability. For credibility, we will meet regularly for peer debriefing sessions to explore one another’s emergent interpretations of the digital stories. For dependability, an audit trail will be maintained in NVivo 14, capturing all codebook iterations. We will ensure confirmability through regular in-person meetings. Prior to completing full data collection or analyzing all data, we will collaborate in-person to ensure our methods are standardized and consensus in our approach is achieved. Lastly, for transferability, we will offer thick description of the Alberta setting, storyteller demographics, and health system features so readers can judge the applicability of results to their own contexts. We will also explicitly interpret findings in light of the repository’s contextual specificity as a publicly curated, provincial collection within a single Canadian health system. Rather than claiming broad generalizability, we aim to support analytic transferability to settings with similar patient engagement infrastructures, storytelling initiatives, and health system contexts.

### Data management

Our data management plan is guided by the Alliance Template for Qualitative Health Sciences Research [32]. All digital stories are housed in the online, open access AHS Patient & Family Storytelling repository [17]. Data collection forms, NVivo project files, reflexive journals, and audit trail materials will be stored on the University of Alberta’s encrypted research server while carrying out the study. For long term preservation, we will store the data collection forms, codebooks, and analytic memos on Open Science Framework (OSF.io) under a CC-BY 4.0 licence. Our public OSF project page where data will be made available can be accessed at https://osf.io/rcun2/. NVivo codebook files (.NVPX) will be exported to preservation-friendly.XML and.CSV formats before deposit. Original videos will not be redistributed because the copyright remains with AHS. Therefore, interested researchers will be directed to the AHS public repository for viewing.

### Timeline

Data collection of field notes from the repository of digital stories is ongoing. We expect to be complete data collection activities by March 31, 2026. We expect our analysis and final report to be prepared by July 31, 2026.

## Discussion

This study contributes methodological knowledge by demonstrating DST as a viable qualitative data source for life course research. Prior studies have used digital stories as interventions to enhance engagement or behaviour change [2,5], but this theory-informed analysis is expected to explore how LCHD principles (cumulative exposures, critical periods, and life transitions) can be used as a lens through which patients, family caregivers, healthcare providers, and policymakers can interpret and learn from a diverse corpus of digital stories. By situating each video within the LCHD framework, the study could illuminate stage-specific patterns, such as how adolescents emphasize identity formation when recounting illness episodes, or how older adults foreground relational losses when describing chronic disease management. Moreover, our work seeks to catalogue structured metadata (life course stage, storyteller role, social context) to create an openly accessible dataset that future researchers can use for secondary analyses on narrative structure, health communication, or media reception. The purpose of cataloguing metadata is not to recreate the AHS repository, but to generate a structured, theory-informed companion dataset for research use. Whereas the original repository functions as a public storytelling platform, our dataset will document analytic descriptors relevant to secondary qualitative inquiry, such as life course stage, health context, and LCHD-related features. This added layer of organization is intended to enhance transparency and reproducibility in how the corpus was analyzed, and to support future comparative or secondary analyses that would otherwise require researchers to independently re-screen the full repository. The examination of reception and perceived impact is also central to this planned work. Previous evaluations suggest viewers of patient-generated videos report heightened empathy, improved recall of health information, and greater readiness for shared decision-making [4,7]. Our insights will seek to inform design principles for health services that wish to integrate DST into patient education, staff orientation, or quality improvement initiatives.

It is important to address the limitation that this study is limited to a data repository from a single Canadian province. However, methodological literature does not denounce the value of a study that is contextualized to a specific place. Qualitative health research typically treats contextual specificity as a strength rather than a threat to validity, emphasizing thick or “thoughtful” description of setting, participants, and phenomena to enable readers to judge relevance to other contexts [33–35]. Thick description goes beyond listing site characteristics to provide nuanced accounts of local organizational routines, sociocultural norms, and policy environments that shape experience and meaning making [34,35]. Such detail underpins transferability understood as case-to-case transfer, resonance, and theoretical engagement, rather than sample-to-population statistical generalizability [33,34]. Furthermore, qualitative findings can inform broader knowledge when higher-order conceptualization is explicit and grounded in richly contextualized data [33,36].

In narrative and digital storytelling research, local health system, sociocultural, and policy contexts are integral to what stories can be told, by whom, and with what effects. Reviews of digital storytelling in health highlight that this method honours local and cultural knowledge and is frequently used with specific cultural or community groups, where stories are co-produced within particular healthcare organizations and service configurations [3,4,37,38]. Context (e.g., clinical vs community setting, country, health system arrangements) is therefore a core analytic category and must be described in sufficient depth to understand how organizational constraints, professional roles, and policy frameworks shape narrative content and uses of stories in practice or knowledge translation [3,4,39]. When drawing broader implications from a single geographic or institutional setting, qualitative research scholars recommend emphasizing resonance and theoretical contribution (e.g., mechanisms of voice, advocacy, or service engagement) while clearly delineating how distinctive local health-system structures, sociocultural dynamics, and policy regimes may limit direct applicability elsewhere [4,34].

### Implications for practice and policy

Findings will be positioned to guide clinical practice by identifying how patient narratives can be used to initiate life course-sensitive conversations (e.g., asking adolescents about identity milestones or older adults about caregiving transitions). For health system leadership, our study will offer an evidence base for investing in DST platforms as vehicles for continuous patient-partnered learning. Among underrepresented groups, it could be recognized that patient engagement work, such as DST, could accelerate health equity goals. At a broader level, illustrating how lived experience narratives align with LCHD constructs will support the inclusion of life course perspectives in health system chronic disease strategies and patient safety frameworks.

## Conclusion

By analyzing the AHS’ life course-spanning repository, this study will generate theoretically grounded recommendations for curating, analyzing, and mobilizing digital stories that are applicable to healthcare organizations, policymakers, community groups, and researchers across Canada and internationally.

## Supporting information

S1 File DSALC Supplementary File 1: Data Collection Form. (PDF)

S2 File DSALC COI. (DOCX)

S3 File DSALC SRQR Checklist. (PDF)

S4 File Appendix. (DOCX)
