# Supplementary material for: Digital storytelling across the life course: Protocol for a theory-based analysis
Source: PLoS One. 2026 Jun 25;21(6):e0332145. doi: 10.1371/journal.pone.0332145 (PMC13298745; doi:10.1371/journal.pone.0332145)
Supplement: S1 File — (PDF) [file pone.0332145.s001.pdf]

Data Collection Form: ***Digital Storytelling Across the Life Course***

Digital Story Title: ____

Medical Condition: ______________________ URL: ____________________

Reviewed by: ______________________ Date: ____________________

Life Course Stage of Person Receiving Care					
**Preconception**	**Infancy**	**Childhood**	**Adolescence**	**Early / Middle adulthood**	**Late / Older adulthood**
☐	☐	☐	☐	☐	☐
**Storyteller's Gender:**			☐ Man	☐ Woman	☐ Other

Life Course Stage of Storyteller *(if not 1st Person Story)*					
**Preconception**	**Infancy**	**Childhood**	**Adolescence**	**Early / Middle adulthood**	**Late / Older adulthood**
☐	☐	☐	☐	☐	☐
**Storyteller's Gender:**			☐ Man	☐ Woman	☐ Other

Place a checkmark in the table below when an LCHD construct is present within the story:

✓	LCHD Construct	Digital Story Element	Analytic Reflection
☐	Health trajectory	Explicit or implicit descriptions of changing health status.	"How does the storyteller describe shifts in wellbeing?"
☐	Sensitive/critical periods	Sharing of moments when exposures outsize impact.	"Does the storyteller highlight an event they see as a turning point?"
☐	Cumulative risk/protective factors	Descriptions of advantages or adversities.	"What social or systemic factors influence this storyteller's narrative?"
☐	Plasticity and adaptive capacity	Evidence of resilience, coping, or biological behavioural adaptation.	"What strategies or supports allowed the narrator to recover?"
☐	Linked lives and intergenerational effects	Influence of caregivers, kin, and communities across generations.	"How does the presence and participation of others shape the narrator's story?"
☐	Timing/environmental context	Policy and healthcare reforms, geographic situation (urban vs. rural).	"Do system-level changes (or the need for) surface in the storyteller's narrative?"

Field Notes:
