# Supplementary material for: Digital storytelling across the life course: Protocol for a theory-based analysis
Source: PLoS One. 2026 Jun 25;21(6):e0332145. doi: 10.1371/journal.pone.0332145 (PMC13298745; doi:10.1371/journal.pone.0332145)
Supplement: S2 File — (DOCX) [file pone.0332145.s002.docx]

**Digital Storytelling Across the Life Course: Protocol for a Theory-Based Analysis**

**Declaration of Conflicting Interest**

Dr. Michael Lang runs two private businesses related to Digital Storytelling. Additional details about the specific activities of these businesses can be found here: [www.mikelangstories.com](http://www.mikelangstories.com) and [www.commonlanguagedst.org](http://www.commonlanguagedst.org).
