# Supplementary material for: Digital storytelling across the life course: Protocol for a theory-based analysis
Source: PLoS One. 2026 Jun 25;21(6):e0332145. doi: 10.1371/journal.pone.0332145 (PMC13298745; doi:10.1371/journal.pone.0332145)
Supplement: S4 File — (DOCX) [file pone.0332145.s004.docx]

**Appendix**

Table 1. Deductive codebook

| **LCHD Construct** | **Digital Story Element** | **Analytic Reflection** |
| --- | --- | --- |
| Health trajectory | Explicit or implicit descriptions of changing health status. | “How does the storyteller describe shifts in wellbeing?” |
| Sensitive/critical periods | Sharing of moments when exposures outsize impact. | “Does the storyteller highlight an event they see as a turning point?” |
| Cumulative risk/protective factors | Descriptions of advantages or adversities. | “What social or systemic factors influence this storyteller’s narrative?” |
| Plasticity and adaptive capacity | Evidence of resilience, coping, or biological behavioural adaptation. | “What strategies or supports allowed the narrator to recover?” |
| Linked lives and intergenerational effects | Influence of caregivers, kin, and communities across generations. | “How does the presence and participation of others shape the narrator’s story?” |
| Timing/environmental context | Policy and healthcare reforms, geographic situation (urban vs. rural). | “Do system-level changes (or the need for) surface in the storyteller’s narrative?” |

Table 2. Pilot Data Collection Inter-rater Reliability

| **LCHD Construct** | **Percent Agreement** | **Scott’s Pi** | **Cohen’s Kappa** | **Krippendorff’s Alpha (nominal)** | N Agreements | N Disagreements | N Cases | N Decisions |
| --- | --- | --- | --- | --- | --- | --- | --- | --- |
| Health trajectory | 40% | -0.429 | 0 | -0.286 | 2 | 3 | 5 | 10 |
| Sensitive/critical periods | 40% | -0.429 | 0 | -0.286 | 2 | 3 | 5 | 10 |
| Cumulative risk/protective factors | 40% | -0.2 | 0.118 | -0.08 | 2 | 3 | 5 | 10 |
| Plasticity and adaptive capacity | 100% | 1 | 1 | 1 | 5 | 0 | 5 | 10 |
| Linked lives and intergenerational effects | 20% | -0.667 | 0 | -0.5 | 1 | 4 | 5 | 10 |
| Timing/environmental context | 80% | 0.6 | 0.615 | 0.64 | 4 | 1 | 5 | 10 |
